# Transcranial Magnetic Stimulation Measures, Pyramidal Score on Expanded Disability Status Scale and Magnetic Resonance Imaging of Corticospinal Tract in Multiple Sclerosis

**DOI:** 10.3390/bioengineering10101118

**Published:** 2023-09-24

**Authors:** Maja Rogić Vidaković, Ana Ćurković Katić, Sanda Pavelin, Antonia Bralić, Una Mikac, Joško Šoda, Ana Jerković, Angela Mastelić, Krešimir Dolić, Anita Markotić, Zoran Đogaš, Nikolina Režić Mužinić

**Affiliations:** 1 Laboratory for Human and Experimental Neurophysiology, Department of Neuroscience, School of Medicine, University of Split, 21000 Split, Croatia; anasuto@gmail.com (A.J.); zdogas@mefst.hr (Z.Đ.); 2 Department of Neurology, University Hospital of Split, 21000 Split, Croatia; spavelin@gmail.com; 3 Department of Interventional and Diagnostic Radiology, University Hospital of Split, 21000 Split, Croatia; antoniascepanovic@gmail.com (A.B.); kdolic79@gmail.com (K.D.); 4 Department of Psychology, Faculty of Humanities and Social Sciences University of Zagreb, 10000 Zagreb, Croatia; umikac@ffzg.hr; 5 Signal Processing, Analysis, Advanced Diagnostics Research and Education Laboratory (SPAADREL), Faculty of Maritime Studies, Department for Marine Electrical Engineering and Information Technologies, University of Split, 21000 Split, Croatia; jsoda@pfst.hr; 6 Department of Medical Chemistry and Biochemistry, School of Medicine, University of Split, 21000 Split, Croatia; angela.mastelic@mefst.hr (A.M.); anita.markotic@mefst.hr (A.M.); nikolina.rezic@mefst.hr (N.R.M.); 7 Department of Radiology, School of Medicine, University of Split, 21000 Split, Croatia

**Keywords:** multiple sclerosis, transcranial magnetic stimulation, motor evoked potential, magnetic resonance imaging, EDSS

## Abstract

Probing the cortic ospinal tract integrity by transcranial magnetic stimulation (TMS) could help to understand the neurophysiological correlations of multiple sclerosis (MS) symptoms. Therefore, the study objective was, first, to investigate TMS measures (resting motor threshold-RMT, motor evoked potential (MEP) latency, and amplitude) of corticospinal tract integrity in people with relapsing-remitting MS (pwMS). Then, the study examined the conformity of TMS measures with clinical disease-related (Expanded Disability Status Scale—EDSS) and magnetic resonance imaging (MRI) results (lesion count) in pwMS. The e-field navigated TMS, MRI, and EDSS data were collected in 23 pwMS and compared to non-clinical samples. The results show that pwMS differed from non-clinical samples in MEP latency for upper and lower extremity muscles. Also, pwMS with altered MEP latency (prolonged or absent MEP response) had higher EDSS, general and pyramidal, functional scores than pwMS with normal MEP latency finding. Furthermore, the RMT intensity for lower extremity muscles was predictive of EDSS functional pyramidal scores. TMS/MEP latency findings classified pwMS as the same as EDSS functional pyramidal scores in 70–83% of cases and were similar to the MRI results, corresponding to EDSS functional pyramidal scores in 57–65% of cases. PwMS with altered MEP latency differed from pwMS with normal MEP latency in the total number of lesions in the brain corticospinal and cervical corticospinal tract. The study provides preliminary results on the correspondence of MRI and TMS corticospinal tract evaluation results with EDSS functional pyramidal score results in MS.

## 1. Introduction

Multiple sclerosis (MS) is an inflammatory autoimmune-mediated disease of the central nervous system (CNS) characterized by white matter demyelinating lesions and neuronal degeneration. The primary pathological event is demyelination, with destruction and loss of axons correlating with a permanent functional deficit [1,2,3,4]. However, pathophysiological correlates and their relation with clinical findings and symptoms are still not elucidated. Therefore, this suggests a need to detect subclinical markers, such as neurophysiological markers, that could identify pathological events involved in individuals with MS at different stages of the disease [5,6,7].

Using navigated transcranial magnetic stimulation (TMS) in probing corticospinal excitability as a marker of functional integrity of the primary motor cortex (M1), corticospinal axonal pathway, and peripheral signaling function to target muscles could help in further understanding the underlying pathophysiological mechanisms of MS. Recent findings proposed that applying TMS as an adjuvant para-clinical instrument could help to identify biomarkers of the MS disease [8], serving as a biomarker of MS disability [9]. An association between the pathophysiological mechanisms of MS (demyelination and axonal loss) and TMS measures (e.g., low amplitudes and prolonged latencies of motor evoked potentials (MEP), increased resting motor threshold (RMT), and increased central motor conduction time) have been reported [6]. Alterations in cortical excitatory and inhibitory processes in MS assessed with TMS are noticeable early in the disease progress, during relapses, and later during the disease progression [6,7,10]. Also, [6,9], from the clinical point of view, diverse quantitative magnetic resonance imaging (MRI) indices (i.e., lesion volume, T2WI value) have been proposed as structural biomarkers of MS. However, the correlations between individual MRI measures and the EDSS have been modest and varying.

The present study aims to (1) investigate TMS measures (resting motor threshold-RMT, motor evoked potential (MEP) latency, MEP amplitude) of corticospinal tract integrity in people with relapsing-remitting MS (pwMS), and (2) to preliminarily assess the conformity of TMS measures with clinical disease-related status (EDSS score) and magnetic resonance imaging (MRI) results (lesion count) in pwMS [11,12,13]. The following hypotheses were settled: (1) pwMS with altered (prolonged or absent) MEP latency will have higher EDSS scores compared to non-clinical samples, (2) pwMS with altered MEP latency will differ from non-clinical samples in the total number of lesions in the brain cortico-spinal and cervical corticospinal tract, and (3) the study will provide preliminary results on correspondence between TMS corticospinal integrity measures (MEP latency findings) and MRI corticospinal tract evaluation (lesion count) with EDSS functional pyramidal scores.

## 2. Materials and Methods

### 2.1. Participants and Study Design

This is a cross-sectional study of 23 pwMS treated with teriflunomide (Aubagio) medication for ≥12 months. None of the pwMS experienced a relapse 3 months before participating in this study. Non-clinical samples from previous studies were used as a control for comparison with pwMS on TMS [14,15,16,17,18,19]. Also, the following exclusion criteria were applied: immunomodulatory drug intake other than teriflunomide, history of diseases of the central or peripheral nervous system (other than relapsing-remitting MS), history of psychiatric diseases, drug or alcohol abuse, and using a TMS screening questionnaire, presence of any contraindication for TMS, for which the safety screening was applied [20].

### 2.2. Clinical Disease-Related Assessment Measures

Neurological examination and medical history included the following measures: the EDSS score and EDSS functional pyramidal score total and for right and left upper and lower extremities [21], MS disease duration, age at onset of MS, drug intake duration, and comorbidities other than MS.

The electroneurographic (ENG) assessment was performed as an additional tool to exclude possible peripheral neurological events in pwMS using the Medelec-Synergy instrument (Oxford Instrument Co., Surrey, UK) [22]. ENG assessment of lower and upper extremities included the following measures for motor nerves (n. medianus, n. ulnaris, n. peroneus, n. tibialis): distal motor latency, compound muscle action potential amplitude, conduction velocity, and F-wave latency; and for sensory nerves (n. medianus, n. ulnaris, n. suralis): sensory nerve action potential amplitude, sensory nerve action potential latency, and conduction velocity.

### 2.3. Radiological Magnetic Resonance Imaging (MRI) Assessment and Image Evaluation

Scans were acquired on a MR 1.5T system (Avanto, Siemens, Medical Systems, Best, Germany) using a 12-channel phased array head coil. The sequences included in the brain scan protocol were 3D T1-weighted images, axial T2-weighted images, and fluid-attenuated inversion recovery (FLAIR) images in the axial and sagittal plane, all used to identify brain lesions. Spinal cord sequences included sagittal T2-weighted images, sagittal turbo inversion recovery magnitude (TIRM) images, and axial T2 med images from C1 to C7 vertebral levels. MRIs were analyzed using Syngo.via software (Siemens Healthcare, Forchheim, Germany) equipped with a screen (1600 × 1200 pixel resolution). MRIs were analyzed by a senior radiology resident (AB) and supervised by an experienced neuroradiologist (KD) with 15 years of experience. Both were blinded to the TMS and EDSS results. Using the T2, FLAIR, TIRM, and T2 med images, specific locations of the corticospinal tract (CST) were visually examined, including subcortical white matter in the primary motor cortex (CST-M1), capsula interna, cerebral peduncles and ventral parts of the midbrain and pons (CST-M2) and ventral and lateral parts of the cervical spinal cord (CST-M3). For each subject, it was checked whether they had a lesion in any listed locations (CST-M1, CST-M2, CST-M3), the number of lesions, and whether the lesion was located on the left or right side. The McDonald’s criteria were consulted for the lesion count for the individual subject [11,12,13]. MRI images were used for the 3D reconstruction of individual brain anatomy (3D optical tracking unit of the manufacturer Polaris ^®^ Vicra) with TMS (Nexstim NBS System 4 of the manufacturer Nexstim Plc., Helsinki, Finland).

### 2.4. Navigated Transcranial Magnetic Stimulation (TMS) Procedure

The magnetic stimulation was delivered using a biphasic single magnetic coil generating a biphasic pulse of a length of 289 µs. The eight-shaped coil with an inner winding diameter of 50 mm and an outer winding diameter of 70 mm was placed tangentially to the subject’s skull over the primary motor cortex (M1). The motor evoked potentials (MEPs) were recorded from the upper extremity muscles (abductor pollicis brevis—APB, abductor digiti minimi—ADM) and lower extremity muscles (tibialis anterior—TA, abductor hallucis—AH) with a pair of self-adhesive surface electrodes (Ambu ^®^ Blue Sensor BR, BR-50-K/12 of manufacturer Ambu A/S) in a belly-tendon montage. Electrodes were attached to the electrode cable of the Nexstim electromyography (EMG) system with a 1.5 mm touch-proof female safety connector (DIN 42-802) and connected to a 6-channel EMG and one common ground EMG amplifier (external module) with TMS-artefact rejection circuitry. The coil was positioned tangentially to the central sulcus to ensure a posterior–anterior current direction over the primary motor cortex (M1). The lowest stimulation intensity used to elicit at least five positive MEP responses out of 10 trials, having peak-to-peak amplitudes larger than 50 µV, was defined as the resting motor threshold (RMT) intensity. When mapping lower extremity muscles (TA, AH) and applying 100% intensity of maximal stimulator output to obtain reliable MEPs, the MEPs with amplitudes slightly lower than 50 µV were collected for the analysis (10 trials). MEP latency and amplitude estimation were performed by a custom-made MATLAB script (R2021a) using an automatic algorithm developed by our research group [23].

### 2.5. Statistical Analysis

Statistical analyses were conducted using Excel 2013 and IBM SPSS Statistics Version 25. Participants’ characteristics were analyzed using descriptive statistics. Since skewness and kurtosis parameters did not indicate significant deviations from a normal distribution for most variables, the parametric statistic was used, except for EDSS, for which we used nonparametric statistics (stated in brackets). Groups were compared with Welch *t*-tests suitable for heterogeneous variances (Mann–Whitney *U* test). These included comparisons of pwMS to non-clinical samples from previous research [14,15,16,17,18,19] and of pwMS with altered MEP latency (prolonged MEP latency or absent MEP latency) with non-altered MEP latency findings (MEP response elicited and non-alterations in MEP observed). Further, correlation analyses were conducted on the whole pwMS sample using Pearson’s r coefficient (or Spearman rank-order correlation ρ). Also, correspondence in classification was tested with McNemar’s test. Finally, a *p* < 0.05 value for the significance was set and considered statistically significant.

## 3. Results

### 3.1. Patient Characteristics

Demographic, clinical, and disease-related characteristics of pwMS are summarized in Table 1. The study sample included 23 pwMS with a mean age of 41.65 (SD = 8.89) years. Most pwMS were women (60.87%), right-handed (91.3%), and with high school education (73.9%). The mean disease duration was 9.39 (SD = 5.73) years, and the median EDSS score was 2.5 (range 0–4). The average duration of immunomodulatory teriflunomide drug intake was 3.6 (SD = 1.66) years. In total, 43.5% of pwMS suffered from other chronic diseases. Average ENG values were in accordance with the referent values indicating no peripheral neurological events [22].

### 3.2. TMS Measures

**Figure 1:**
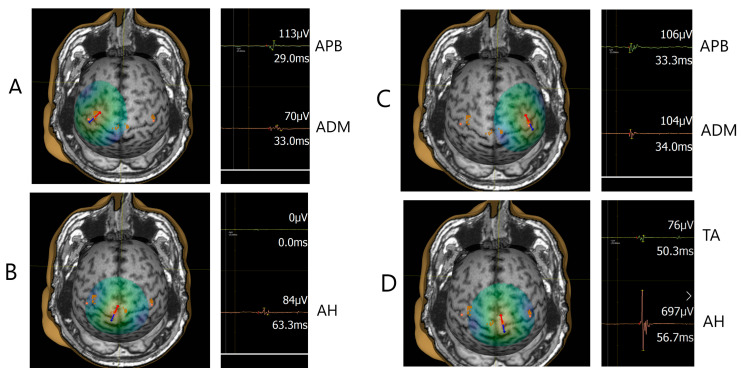
Mapping of the M1 for the representation of the upper (**A**,**C**) and lower extremities (**B**,**D**) and registration of single trial MEP response from the muscles of the upper (APB, ADM) and lower extremities (TA, AH) in the male subject (No. 3). The arrow indicates the position of the coil perpendicular to the central sulcus and point of stimulation. Values of the MEP peak-to-peak amplitude (µV) and MEP latency (ms) are indicated for each MEP trial.

**Figure 2:**
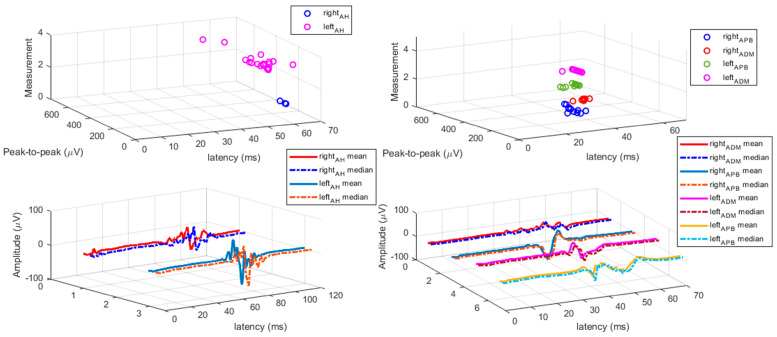
All MEP responses from upper and lower extremity muscles for male subject No. 3. Left: MEP responses elicited for lower extremity AH muscle; left upper: MEP responses for left and right AH muscle; left below: mean and median for left and right MEPs for AH muscle. Right: MEP responses elicited for upper extremity APB and ADM muscle; right upper: MEP responses for left and right APB and ADM muscle; right below: mean and median for left and right MEPs for APB and ADM muscle. MEP peak-to-peak amplitude is expressed in microvolts (µV), and MEP latency in milliseconds (ms).

**Figure 3:**
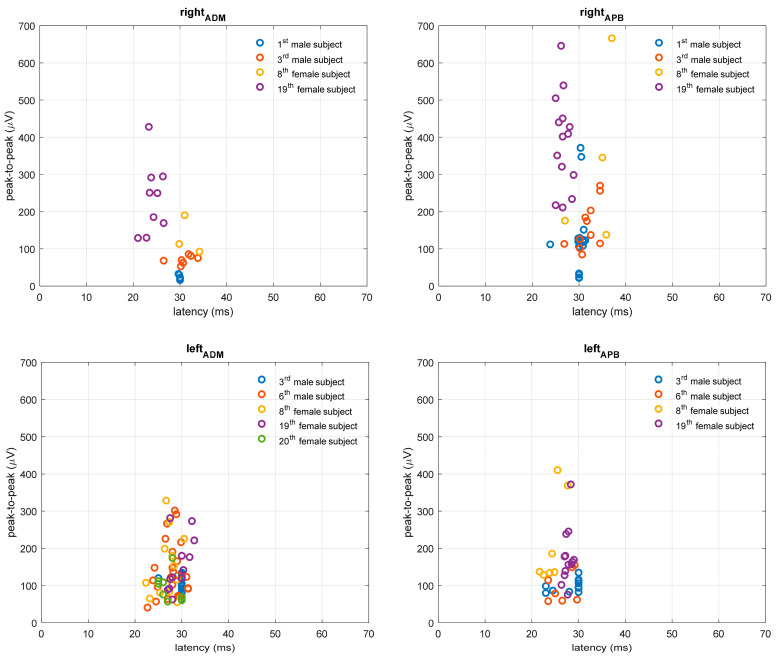
MEP responses for upper extremity APB and ADM muscles in pwMS with altered TMS finding. MEP peak-to-peak amplitude is expressed in microvolts (µV), and latency in milliseconds (ms).

**Figure 4:**
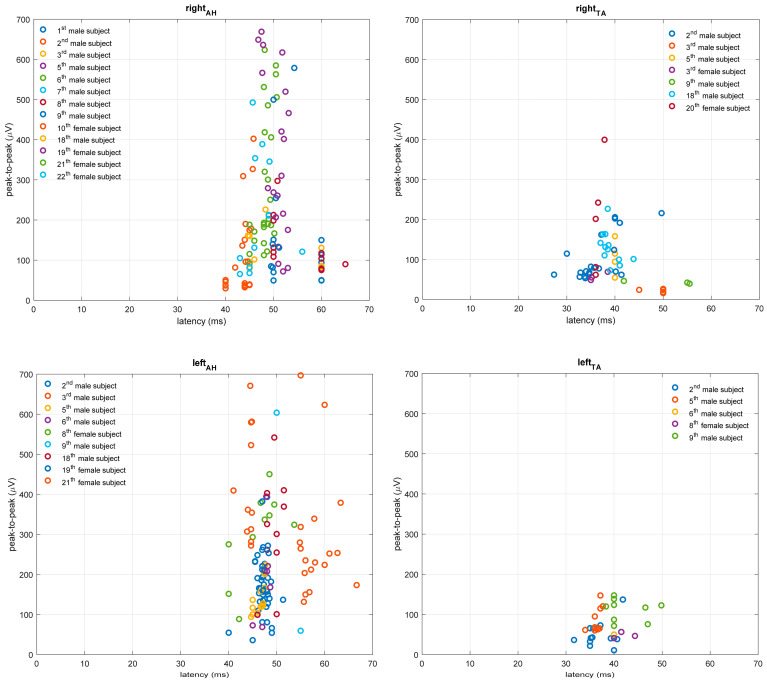
MEP responses for lower extremity TA and AH muscles in pwMS with altered TMS finding. MEP peak-to-peak amplitude is expressed in microvolts (µV), and MEP latency in milliseconds (ms).

Fifteen out of 23 pwMS had altered MEP latency (prolonged or absent MEP) (65.2%), and eight subjects had normal MEP latency findings (MEP responses were elicited/present). Table 2 presents TMS measures (RMT, MEP latency, and MEP amplitude) for upper and lower extremity muscles. It can be observed that a significant difference was found for RMT between left and right hemisphere stimulation of APB (*t*(22) = 2.87, *p* = 0.009), which was higher for the right hemisphere (*M*_right_ = 47.00, *M*_left_ = 40.26). The results of TMS measures (RMT, MEP latency, and MEP amplitude) for upper and lower extremity muscles for individual pwMS subjects divided into pwMS samples with altered MEP findings and pwMS with normal (non-altered) MEP findings are presented in Supplementary Table S1. When comparing TMS measures of the pwMS sample in the present study and non-clinical samples (Table 3) [14,15,16,17,18,19], significantly prolonged MEP latencies were found for all muscles (left: *t*_APB_(23) = 4.94, *p* < 0.001; *t*_ADM_(24) = 6.34, *p* < 0.001; *t*_TA_(16) = 6.12, *p* < 0.001; *t*_AH_(32) = 6.52, *p* < 0.001; right: *t*_APB_(23) = 6.14, *p* < 0.001; *t*_ADM_(24) = 7.15, *p* < 0.001; *t*_TA_(15) = 7.64, *p* < 0.001; *t*_AH_(30) = 4.96, *p* < 0.001) in the pwMS sample in the current study. Further, significantly prolonged MEP latencies were also found for all muscles when comparing non-clinical samples with pwMS with altered MEP latency findings (Table 3). Figure 1 presents single trial MEP latencies from upper and lower extremity muscles for an MS subject (No. 3) having four lesions in the corticospinal pathway (CST-M3), two lesions on the left and two lesions on the right with the positioning of positive, stimulating spots over the M1 for the representation of the upper (Figure 1A, C) and lower extremities (Figure 1B, D). Figure 2 presents all MEP latency trials from upper and lower extremity muscles for subject No. 3. Figure 3 depicts MEP responses from upper extremity muscles, and Figure 4 presents MEP responses from lower extremity muscles for all pwMS with altered MEP findings.

### 3.3. TMS, EDSS, and MRI Correspondence Results

PwMS with altered MEP finding (prolonged MEP latency or absent MEP) had higher EDSS score [Median _TMS-A_ = 3.5 (range 0–4), Median _TMS-N_ = 0.5(0–2.5), Mann–Whitney *U* = 19.5, *p* = 0.008] and EDSS functional pyramidal score [Median _TMS-A_ = 3.0(0–3.5); Median _TMS-N_ = 0.5(0–2) Mann–Whitney *U* = 18, *p* = 0.005], as well as for both lower extremities (right/left: Mann–Whitney *U* = 15.5/27, *p* = 0.002/0.034), but not upper extremities (right/left: Mann–Whitney *U* = 51/40, *p* = 0.591/0.213). Results indicate a significant correlation between MEP latency of lower extremity AH (ρ = 0.548, *p* < 0.01) and TA (ρ = 0.543, *p* < 0.05) muscle of the right leg and EDSS functional pyramidal score for the right leg. Also, a significant correlation was observed between RMT intensity for mapping representations for lower extremity TA and AH muscles over the right hemisphere and EDSS score (TA/AH, ρ = 0.615, *p* < 0.01; AH, ρ = 0.642, *p* < 0.01), EDSS functional pyramidal score (TA, ρ = 0.593, *p* < 0.01; AH, ρ = 0.654, *p* < 0.01), EDSS functional pyramidal score for the right leg (TA, ρ = 0.509, *p* < 0.05; AH, ρ = 0.560, *p* < 0.01) and EDSS functional pyramidal score for the left leg (TA, ρ = 0.615, *p* < 0.01; AH, ρ = 0.578, *p* < 0.01). Next, a significant correlation was observed between RMT intensity for mapping representations for AH muscle over the left hemisphere and EDSS score (ρ = 0.462, *p* < 0.05), EDSS functional pyramidal score (ρ = 0.480, *p* < 0.05), and EDSS functional pyramidal score for the right leg (ρ = 0.514, *p* < 0.05). PwMS with altered MEP latency differed from those with non-altered MEP latency finding on the total number of lesions in the brain corticospinal (*t*(11) = 3.05, *p* = 0.01) and in the cervical corticospinal (right: *t*(19) = 2.32, *p* = 0.03; left: *t*(19) = 2.23, *p* = 0.04) tract. A number of lesions in the right corticospinal tract (CST-M3) correlated with the EDSS functional pyramidal score for the right leg (ρ = 0.425, *p* < 0.05). Table 4 presents the lesion count according to McDonald’s criteria and MRI lesion evaluation of the corticospinal tract.

The correspondence of EDSS pyramidal functional scoring of each extremity with classifications based on TMS corticospinal tract integrity and MRI on corticospinal tract evaluation is presented in Table 5. It can be observed that TMS findings classified pwMS as the same as EDSS pyramidal functional score in 70–83% of cases and MRI in 57–65% of cases. Moreover, McNemar’s test indicated TMS and MRI showed equal correspondence with the EDSS pyramidal functional score for the right arm’s muscles and for the lower extremity muscles. For the left arm muscles, McNemar’s test indicated significantly better correspondence of TMS (compared to MRI) with EDSS score (χ2 = 3.12, *p* = 0.047). Therefore, TMS was more accurate (83%) for the left arm in replicating the EDSS pyramidal functional score than MRI (57%). The correspondence of EDSS general/pyramidal with classifications based on TMS corticospinal tract integrity and MRI on corticospinal tract evaluation is presented in Table 6.

## 4. Discussion

In this cross-sectional study, we found prolongations in MEP latency or absence of MEP response in lower and upper extremity muscles in 65.2% of pwMS. The study results correspond to previous findings on the correlation between EDSS scores and TMS measures (MEP latency and RMT intensity) [6,9]. The association between neurophysiological TMS measures of cortical excitability and clinical results (EDSS) could indicate a predominant role of white matter lesions in the pathogenesis of these changes, especially of the corticospinal pathway and corpus callosum [6]. Further, the present study provides preliminary results on correspondence between TMS corticospinal integrity measures (MEP latency findings) and MRI corticospinal tract evaluations (lesion count) with EDSS functional pyramidal scores.

From the clinical point of view, MRI is a sensitive test for diagnosing and assessing disease progression in pwMS and is often used to monitor therapeutic efficacy. However, the poor association between conventional MRI measures of tissue damage, such as T1-weighted or T2-weighted lesion load, and clinical disability measured with EDSS has been reported previously [24,25,26,27,28]. On the other hand, it is pointed out that TMS is not used as a standard technique in clinical assessment of pwMS and is currently recommended to monitor the integrity of the corticospinal tract in clinical follow-up [29]. The findings from the present study point to the practical value of TMS mapping of the corticospinal tract integrity and estimating the EDSS functional pyramidal score, in addition to the overall EDSS score.

Finally, there are limitations in the present study. The control data (non-clinical samples) from previous reports were used [14,15,16,17,18,19,30]. Due to the unavailability of diffusion tensor imaging (DTI) at our institution, fiber tractography, and automated atlases [31,32], we were limited in the identification and reconstruction of the corticospinal tract by applying MRI lesion count [28]. The MEP peak-to-peak amplitude and RMT could not be properly compared with non-clinical samples, since most of the studies reported MEP latency average results (including mean, standard deviation, and the number of subjects). Fourth, a larger sample would allow more power for our conclusions, i.e., less chance for so-called Type I error and additional statistical analyses. Furthermore, although MEP latency measures corresponded with clinical scores (EDSS), further validation of MEP measurements is needed regarding their validity, reliability, and sensitivity in longitudinal study before being routinely used in clinical trials in pwMS [33]. Also, EDSS is a standard tool for functional disability inspection applied by a physician and requires an in-person assessment and suffers from having high inter- and intra-rater variability, particularly at the lower disability levels [34]. In our study, the EDSS evaluation of pwMS was performed by an experienced physician for all patients to avoid interrater variability. Other functional tests like the 6 min walk-test (6MWT) or 10 m walk-test (10MWT), could be assessed with EDSS to evaluate clinically relevant disability status with TMS mapping of the corticospinal tract integrity in pwMS [5].

The advantage of the present study is the application of e-field navigated TMS in evaluating corticospinal tract integrity and its correspondence with the EDSS functional pyramidal score (general and for each extremity) in pwMS. Recent reports [35] suggest the use of e-field navigated TMS to improve the accuracy of corticospinal tract integrity testing by providing more objective correspondence of neurophysiological (e-field navigated TMS) and clinical (EDSS and MRI) classifications.

## 5. Conclusions

PwMS with prolonged or absent MEP response had higher EDSS, general and pyramidal, functional scores than pwMS with normal MEP latency findings. Further, RMT intensity for lower extremity muscles was predictive of EDSS functional pyramidal scores. The present study provides preliminary findings on the similar correspondence of TMS and MRI evaluations of corticospinal tract integrity results with EDSS pyramidal functional score classifications. The study findings point to the clinical value of the neurophysiological TMS methodology in evaluating corticospinal tract integrity as an additional armamentarium to EDSS, a standard clinical, functional disability instrument. Ultimately, TMS has the potential advantage of being a continuous variable compared to the discrete nature of EDSS; however, the usefulness of TMS mapping of corticospinal tract integrity in this regard can only be determined through longitudinal studies.

## Figures and Tables

**Table 1:** Demographic, clinical and disease-related characteristics.

Demographic and Clinical Characteristics	N	%	Mean (SD) Median (IQR)
Mean age in years			41.6 (8.8)
Female/Male	14/9	60.9/39.1	
Education (elementary school/high school/undergraduate university study/graduate university study) Right hand dominance (%)	2/17/1/3 21	8.7/73.9/4.4/13.0 91.3	
Mean height (cm)			175 (10.2)
Mean weight (kg)			77.9 (19.0)
Mean BMI (kg/m^2)			25.1 (3.7)
Disease-related characteristics			
Mean age at onset of MS in years			31.7 (10.9)
Mean MS disease duration in years			9.3 (5.7)
Mean number of relapses from MS diagnosis			4.0 (3.7)
Mean Teriflunomide intake duration in years			3.6 (1.6)
Other chronic diseases, not MS (yes/no)	10/13	43.5/56.5	
Median EDSS score			2.5 (3.5)
Median EDSS functional pyramidal score			2.0 (3)
Median EDSS functional pyramidal score right leg			1.0 (2.5)
Median EDSS functional pyramidal score left leg			1.0 (2.5)
Median EDSS functional pyramidal score right arm			0 (0)
Median EDSS functional pyramidal score left arm			0 (0)

**Table 2:** Results of TMS measures (RMT, MEP latency, and MEP amplitude) for upper and lower extremity muscles.

TMS			Altered MEP (N = 15)			Non-Altered MEP (N = 8)			All pwMS subjects (N = 23)		
	Muscle	Measure	*M*	*SD*	Absent MEP (%)	*M*	*SD*	Absent MEP (%)	*M*	*SD*	Absent MEP (%)
LEFT hemisphere stimulation	APB	RMT %	41.4	10.2	0	38.0	7.60	0	40.2	9.4	0
	APB	MEP L (ms)	25.14	4.1	0	22.0	1.42	0	24.0	3.7	0
	APB	MEP A (µV)	318.9	192.7	0	195.6	88.65	0	276.0	172.4	0
	ADM	RMT %	44.5	13.7	0	38.0	6.97	0	42.2	12.1	0
	ADM	MEP L (ms)	24.7	3.5	0	22.2	1.03	0	23.9	3.1	0
	ADM	MEP A (µV)	167.1	102.0	0	154.3	52.12	0	162.7	86.7	0
	TA	RMT %	85.6	15.6	6.67	62.8	28.09	0	81.1	15.5	4.3
	TA	MEP L (ms)	39.3	7.8	33.33	27.3	11.29	0	36.0	7.0	21.7
	TA	MEP A (µV)	95.3	52.3	33.33	95.4	70.49	0	101.5	54.2	21.7
	AH	RMT %	84.0	15.0	0	67.1	13.21	0	78.1	16.3	0
	AH	MEP L (ms)	49.0	5.0	6.67	43.2	1.83	0	46.9	5.0	4.3
	AH	MEP A (µV)	208.9	141.1	6.67	331.4	109.50	0	253.5	141.3	4.3
RIGHT hemisphere stimulation	APB	RMT %	49.8	16.4	0	41.6	6.1	0	47.0	14.1	0
	APB	MEP L (ms)	24.5	3.1	6.6	22.6	1.2	0	23.8	2.7	4.3
	APB	MEP A (µV)	178.4	83.4	6.6	339.1	373.9	0	232.0	229.1	4.3
	ADM	RMT %	52.2	16.6	0	42.5	5.9	0	48.8	14.4	0
	ADM	MEP L(ms)	25.1	2.9	0	22.1	1.2	0	24.0	2.8	0
	ADM	MEP A (µV)	210.3	134.1	0	192.3	164.9	0	204.1	142.0	0
	TA	RMT %	89.5	11.7	0	65.1	11.8	0	82.5	16.0	0
	TA	MEP L (ms)	35.5	4.6	26.6	32.1	1.0	0	34.2	4.0	17.3
	TA	MEP A (µV)	147.9	184.9	26.6	134.8	80.3	0	143.0	150.7	17.3
	AH	RMT %	87.2	12.9	6.6	63.8	10.8	0	78.7	16.5	4.3
	AH	MEP L (ms)	46.6	5.2	20.0	42.2	0.7	0	44.8	4.5	13.0
	AH	MEP A (µV)	260.1	114.8	20.0	557.2	336.8	0	378.9	267.8	13.0

Abbreviations: MEP L = MEP Latency; MEP A = MEP Amplitude; RMT = resting motor threshold; altered MEP (prolonged MEP latency finding); absent MEP (MEP response could not be elicited in the muscle); M = mean; SD = standard deviation; ms = millisecond; µV = microvolt.

**Table 3:** Comparison of results of TMS measures between pwMS in the present study and non-clinical samples.

		LEFT Hemisphere Stimulation						RIGHT Hemisphere Stimulation					
Muscle		APB		ADM		TA	AH	APB		ADM		TA	AH
Measure		RMT (%)	MEP L (ms)	RMT (%)	MEP L (ms)	MEP L (ms)	MEP L (ms)	RMT (%)	MEP L (ms)	RMT (%)	MEP L (ms)	MEP L (ms)	MEP L (ms)
Current study All pwMS subjects N = 23	M	40.2	24.0	42.2	23.9	36.0	46.9	47.0	23.8	48.8	24.0	34.2	44.8
	SD	9.4	3.70	12.1	3.1	7.0	5.0	14.1	2.7	14.4	2.8	4.0	4.5
Current study pwMS –altered MEP N = 15	M	41.4	25.1	44.5	24.7	39.3	49.0	49.8	24.5	52.2	25.1	35.5	46.6
	SD	10.2	4.1	13.7	3.5	7.8	5.0	16.4	3.1	16.6	2.9	4.6	5.2
Published studies Non-clinical sample		Triggs et al. [14]	Eisen and Shtybel [15]	Macdonell and Donnan [16]	Claus [17]	Cantone et al. [18]	Osei-Lah and Mills [19]	Triggs et al. [14]	Eisen and Shtybel [15]	Macdonell and Donnan [16]	Claus [17]	Cantone et al. [18]	Osei-Lah and Mills [19]
	M	41.6	20.2	49	19.7	25.5	39.1	38.5	20.2	49	19.7	26.5	39.1
	SD	7	1.6	8	1.0	2.2	2.5	6	1.6	8	1.0	2.2	2.5
	N	30	150	20	54	487	20	30	150	20	54	487	20
Comparisons with the current study All pwMS subjects	t	−0.5	4.94	−2.18	6.34	6.12	6.52	2.70	6.14	−0.04	7.15	7.64	4.96
	df	39	23	38	24	16	32	28	23	35	24	15	30
	*p*	0.33	<0.001	0.04 *	<0.001	<0.001	<0.001	0.01 *	<0.001	0.40	<0.001	<0.001	<0.001
Comparisons with the current study pwMS-TMS altered finding	t	−0.05	4.61	−1.12	5.55	5.26	6.84	2.59	5.11	0.70	7.00	6.12	4.67
	df	21	14	21	15	8	18	16	14	19	15	9	14
	*p*	0.39	<0.001	0.21	<0.001	<0.001	<0.001	0.02 *	<0.001	0.30	<0.001	<0.001	<0.001

Note: * *p* < 0.05.

**Table 4:** Number of lesions according to McDonald criteria and lesion evaluation of corticospinal tract.

	pwMS (N = 23)	McDonald	Corticospinal Tract	Corticospinal Tract Right	Corticospinal Tract Left
Altered MEP	1.	44	3	2	1
	2.	38	3	2	1
	3.	14	4	2	2
	4.	16	3	0	3
	5.	23	2	0	2
	6.	5	1	0	1
	7.	49	3	2	1
	8.	33	4	2	2
	9.	6	2	2	0
	10.	13	2	1	1
	11.	8	1	0	1
	12.	50	2	0	2
	13.	50	2	1	1
	14.	20	6	3	3
	15.	13	4	3	1
Non -altered MEP	16.	19	0	0	0
	17.	90	0	0	0
	18.	18	0	0	0
	19.	10	0	0	0
	20.	42	5	2	3
	21.	34	0	0	0
	22.	8	0	0	0
	23.	12	0	0	0
M		26.73	2.04		
SD		20.54	1.79		

**Table 5:** Correspondence of EDSS pyramidal functional score classifications with classifications based on TMS and MRI corticospinal tract results for lower and upper extremity muscles.

pwMS (N = 23)	RIGHT LEG			LEFT LEG			RIGHT ARM			LEFT ARM		
	TMS	MRI	EDSS	TMS	MRI	EDSS	TMS	MRI	EDSS	TMS	MRI	EDSS
1.	1	3	3	0	0	1	1	3	0	0	0	0
2.	1	0	1	1	3	1	0	0	0	0	3	0
3.	1	2	3	1	2	1	1	2	3	1	2	3
4.	1	0	0	1	3	0	0	0	0	0	3	0
5.	1	0	2	1	2	1	0	0	0	1	2	0
6.	1	0	2	1	2	2	0	0	0	0	2	0
7.	1	1	2.5	1	2	2.5	1	1	1	1	2	1
8.	1	2	3.5	1	2	3.5	0	2	0	0	2	0
9.	1	2	3	0	0	0	0	2	0	0	0	0
10.	1	1	3	0	1	3	0	1	0	0	1	0
11.	1	1	1	1	0	1	0	1	1	0	0	1
12.	1	1	2	1	1	3	1	1	0	1	1	0
13.	1	1	1	1	1	3	0	1	0	1	1	2
14.	1	4	0	1	4	0	0	4	0	0	4	0
15.	1	1	2	1	3	3	0	1	1	0	3	1
16.	0	0	0	0	0	0	0	0	0	0	0	0
17.	0	0	1	0	0	1	0	0	0	0	0	0
18.	0	0	1	0	0	2	0	0	0	0	0	0
19.	0	0	0	0	0	0	0	0	0	0	0	0
20.	0	2	0	0	3	0	0	2	0	0	3	0
21.	0	0	1	0	0	1	0	0	0	0	0	0
22.	0	0	0	0	0	0	0	0	1	0	0	0
23.	0	0	0	0	0	0	0	0	0	0	0	0
TMS % of the corresponding classification *	78%			70%			78%			83%		
MRI % of the corresponding classification *	65%			65%			61%			57%		
χ^2^	0.8			0			1.125			3.125		
*p*	0.298			1			0.214			0.047 *		

TMS (1) denotes subjects with altered MEP findings (prolonged MEP or absent MEP response), and TMS (0) denotes subjects with non-altered MEP findings; EDSS (Expanded Disability Status Scale) functional pyramidal score for each extremity; MRI (magnetic resonance imaging) number of lesion(s) in the corticospinal tract. Note: for McNemar’s test, the coding was as follows: subjects with MRI lesion 1 or ≥1 were coded as 1, and subjects with no MRI lesions in the corticospinal tract were coded as 0. * *p* < 0.05

**Table 6:** Correspondence of EDSS classifications (general and pyramid score) with classifications based on TMS and MRI corticospinal tract results.

pwMS (N = 23)	Right Leg		Left Leg		Right Arm		Left Arm		EDSS General/Pyramid Score	EDSS General	EDSS Pyramid
	TMS	MRI	TMS	MRI	TMS	MRI	TMS	MRI			
1.	1	3	0	0	1	3	0	0	1	3.5	3
2.	1	0	1	3	0	0	0	3	1	1.5	1
3.	1	2	1	2	1	2	1	2	1	3.5	3
4.	1	0	1	3	0	0	0	3	0	0	0
5.	1	0	1	2	0	0	1	2	1	2.5	2
6.	1	0	1	2	0	0	0	2	1	2.5	2
7.	1	1	1	2	1	1	1	2	1	3.5	2.5
8.	1	2	1	2	0	2	0	2	1	4	3.5
9.	1	2	0	0	0	2	0	0	1	3	3
10.	1	1	0	1	0	1	0	1	1	3.5	3
11.	1	1	1	0	0	1	0	0	1	1	1
12.	1	1	1	1	1	1	1	1	1	3.5	3
13.	1	1	1	1	0	1	1	1	1	3.5	3
14.	1	4	1	4	0	4	0	4	0	0	0
15.	1	1	1	3	0	1	0	3	1	4	3
16.	0	0	0	0	0	0	0	0	0	0	0
17.	0	0	0	0	0	0	0	0	1	2	1
18.	0	0	0	0	0	0	0	0	1	2	2
19.	0	0	0	0	0	0	0	0	0	0	0
20.	0	2	0	3	0	2	0	3	0	0	0
21.	0	0	0	0	0	0	0	0	1	2.5	1
22.	0	0	0	0	0	0	0	0	1	1	1
23.	0	0	0	0	0	0	0	0	0	0	0
Corresponding classification	74%	61%	61%	57%	44%	61%	48%	57%			
χ^2^	1.88		0.38		0		0				
*p*	0.17		0.54		1		1				

Note: EDSS general and EDSS pyramid have equal corresponding scores: 1 denotes subjects with EDSS≥1, and 0 denotes subjects with EDSS = 0; MRI–1 denotes subjects with MRI lesion 1 or ≥ 1, and 0 denotes no MRI lesions in the corticospinal tract.

## Data Availability

Data are available with a granted proposal upon reasonable request.

## Supplementary Materials

The following supporting information can be downloaded at: https://www.mdpi.com/article/10.3390/bioengineering10101118/s1, Table S1: TMS measures (RMT, MEP latency, and MEP amplitude) for individual pwMS subjects.
