# Supplementary material for: Transcranial Magnetic Stimulation Measures, Pyramidal Score on Expanded Disability Status Scale and Magnetic Resonance Imaging of Corticospinal Tract in Multiple Sclerosis
Source: Bioengineering (Basel). 2023 Sep 24;10(10):1118. doi: 10.3390/bioengineering10101118 (PMC10604490; doi:10.3390/bioengineering10101118)
Supplement: Supplementary file 1 [file bioengineering-10-01118-s001.zip › bioengineering-2620300-supplementary.pdf]

Table S1. TMS measures (RMT, MEP latency, and MEP amplitude) for individual pwMS subjects.

		LEFT hemisphere stimulation											
		APB			ADM			TA			AH		
	pwMS	RMT %	MEP Latency (ms)	MEP Amplitude (μV)	RMT %	MEP Latency (ms)	MEP Amplitude (μV)	RMT %	MEP Latency (ms)	MEP Amplitude (μV)	RMT %	MEP Latency (ms)	MEP Amplitude (μV)
Pwms TMS-A	1	44	29,9	126,8	76	29,9	24,6	100	*	*	100	60,0	91,4
	2	45	25,3	390,6	45	24,3	419,1	67	33,5	77,4	70	44,3	194,82
	3	36	31,7	160,6	42	30,7	82,0	79	47,0	17,8	79	55,3	95,59
	4	51	26,6	288,6	51	25,8	112,0				87	51,9	242,43
	5	24	22,8	113,8	24	23,4	130,2	59	36,2	57,4	55	48,6	484,71
	6	40	20,7	781,3	40	23,1	127,6	100	*	*	92	47,7	334,09
	7	44	33,7	331,4	44	31,6	132,1	100	56,0	45,2	100	55,0	139,43
	8	26	24,3	501,5	26	24,2	199,7	74	39,0	129,5	74	50,4	254,46
	9	49	24,4	132,4	55	23,7	99,1	*	*	*	100	45,0	38,62
	10	37	19,7	110,2	37	18,6	84,2	100	*	*	100		*
	11	33	22,6	160,8	33	23,2	235,6	79	37,0	184,8	79	45,8	145,67
	12	53	26,6	389,5	53	24,0	236,6	100	*	*	78	49,3	492,05
	13	49	22,1	465,6	51	22,5	126,0	93	40,8	144,0	90	43,3	132,06
	14	31	25,9	499,3	31	24,6	320,9	65	31,7	105,9	59	47,0	184,72
	15	60	20,3	331	60	21,5	177,5	98	32,3	95,2	98	43,0	95,49
Pwms TMS-N	1	36	20,4	90,0	36	20,1	151,5	0	0	0	57	40,1	370,64
	2	29	21,4	185,1	29	22,1	179,0	74	31,7	94,9	72	45,0	427,24
	3	35	24,5	289,1	37	23,1	111,3	69	31,7	74,6	65	41,9	344,33
	4	38	21,6	350,3	37	21,7	104,6	81	30,1	71,6	70	43,0	138,32
	5	48	23,0	183,9	48	23,1	130,3	81	33,3	129,0	78	42,6	370,47
	6	34	20,4	105,3	34	21,7	269,2	50	32,3	245,2	50	44,2	341,03
	7	33	22,8	146,4	34	22,7	137,4	61	26,3	83,8	55	43,0	460,55
	8	51	21,9	214,9	49	23,2	150,6	87	33,3	64,3	90	45,8	199,24
	**M**	**40,26**	**24,07**	**276,07**	**42,26**	**23,91**	**162,70**	**81,14**	**36,05**	**101,57**	**78,17**	**46,96**	**253,52**
	**SD**	**9,42**	**3,70**	**172,45**	**12,10**	**3,12**	**86,77**	**15,58**	**7,09**	**54,23**	**16,37**	**5,01**	**141,33**
	No MEP response							1	5	5		1	1
	% no response							4,35%	21,74%	21,74%		4,35%	4,35%

		**RIGHT hemisphere stimulation**											
		**APB**			**ADM**			**TA**			**AH**		
	pwMS	**RMT %**	**MEP Latency (ms)**	**MEP Amplitude (µV)**	**RMT %**	**MEP Latency (ms)**	**MEP Amplitude (µV)**	**RMT %**	**MEP Latency (ms)**	**MEP Amplitude (µV)**	**RMT %**	**MEP Latency (ms)**	**MEP Amplitude (µV)**
**pwMS MEP altered**	1	42	23,0	119,5	42	22,9	204,5	94	32,5	187,0	94	41,6	254,39
	2	48	27,6	63,3	49	25,4	208,1	87	36,6	51,6	87	47,2	152,31
	3	37	30,0	99,7	37	30,0	104,5	80	-	-	80	58,1	284,57
	4	44	27,0	245,8	44	25,0	93,8	82	36,4	85,9	82	46,4	142,63
	5	31	26,5	96,5	31	27,8	124,0	62	40	49,8	54	47,1	176,83
	6	48	23,6	96,7	49	24,2	106,4	100	*	*	N*	*	*
	7	54	25,0	288,2	54	26,9	152,4	100	41,9	47,9	84	46,3	352,88
	8	45	25,7	350,6	45	25,3	106,4	87	42,4	111,4	89	52,5	331,58
	9	57	18,9	182,9	57	19,6	514,6	96	28,7	61,7	96	40,0	44,06
	10	49	20,8	197,5	49	21,5	234,3	99	31,5	122,6	99	39,5	410,82
	11	35	22,7	261,1	35	24,5	433,1	73	32,7	659,9	73	49,2	306,69
	12	53	27,6	177,3	53	29,3	156,4	100	*	*	100	46,8	241,82
	13	100	*	*	91	27,1	95,2	100	*	*	100	*	*
	14	40	23,5	166,5	83	25,3	398,8	83	32,7	101,4	83	44,2	423,03
	15	65	20,8	152,3	65	21,1	222,07	100	*	*	100	*	*
**pwMS MEP non altered**	1	37	21,7	98,6	43	21,1	41,6	-	-	-	56	42,7	271,44
	2	43	24,1	209,9	43	23,3	566,3	-	-	-	79	42,2	647,4
	3	42	23,8	521,4	43	22,7	107,5	75	31,9	73,1	72	41,5	551,48
	4	37	22,5	161.4	37	20,4	129,3	64	30,1	100,8	60	41,6	1308,72
	5	51	23,6	1118,4	51	24,2	172,6	62	32,9	217,5	62	42,1	500,49
	6	39	20,9	71,9	39	21,7	215,2	54	32,5	254,9	55	41,7	435,99
	7	34	23,2	172,6	34	22,2	233,0	53	32,3	92,4	50	42,1	532,62
	8	50	21,3	181,3	50	21,1	73,3	83	32,9	70,0	77	43,9	209,45
**M**		**47,0**	**23,8**	**232,0**	**48,8**	**24,0**	**204,1**	**82,5**	**34,2**	**143,0**	**78,7**	**44,8**	**378,96**
**SD**		**14,1**	**2,7**	**229,1**	**14,4**	**2,8**	**142,0**	**16,0**	**4,0**	**150,7**	**16,5**	**4,5**	**267,82**
No MEP response			1	1					4	4	1	3	3
% no response			4,35%	4,35%					17,39%	17,39%	4,35%	13,04%	13,04%

*Abbreviations: APB –Abductor Pollicis Brevis ; ADM – Abductor Digiti Minimi ; TA – Tibialis Anterior; AH – Abductor Hallucis; RMT % –Resting Motor Threshold; SD – standard deviation; pwMS -people with multipe sclerosis; pwMS with altered MEP findings (prolonged MEP latency or absent MEP response); pwMS with non altered MEP findings (MEP response induced); * -no MEP response; "-" technical error.*
